# Graphene Coatings for Durable and Robust Resistance to Caustic Corrosion of Nickel

**DOI:** 10.3390/nano16040265

**Published:** 2026-02-18

**Authors:** Tanuj Joshi, R. K. Singh Raman, Yiannis Ventikos, Saad Al-Saadi, Anthony De Girolamo

**Affiliations:** 1 Department of Mechanical and Aerospace Engineering, Monash University, Clayton 3800, Australia; tanuj.joshi@monash.edu (T.J.); yiannis.ventikos@monash.edu (Y.V.); 2 Department of Chemical and Biological Engineering, Monash University, Clayton 3800, Australia; saad.al-saadi@monash.edu (S.A.-S.); anthony.degirolamo@monash.edu (A.D.G.)

**Keywords:** 0.5 M NaOH, graphene coatings, corrosion resistance, multilayer graphene (MLG), time-dependent electrochemical test

## Abstract

Nickel is widely deployed in caustic service, yet its native Ni(OH)_2_/NiOOH passive film raises concerns for long service life. Graphene has emerged as a promising corrosion barrier; however, its long-term durability in strongly alkaline media remains largely unexplored. The extended exposure period in a highly caustic solution is a novel aspect of the present work, distinguishing it from previous studies that predominantly examined short-term exposures or focused on neutral and acidic environments. Here, we present the systematic assessment of low-pressure CVD-grown multilayer graphene (MLG) coatings on Ni in highly caustic (0.5 M NaOH) for up to 80 days. Two architectures, a conformal, robust MLG coating (Gr_Ni) and a less robust film (Gr_Ni_DF), were benchmarked against bare Ni. PDP and EIS reveal that Gr_Ni initially delivers nearly 2 orders of magnitude enhancement, as evidenced by the low frequency impedance, accompanied by a broad, high-fidelity capacitive plateau; the impedance still maintains 1.3–1.5 orders of magnitude superior after prolonged exposure. In contrast, Gr_Ni_DF undergoes progressive degradation, affording a modest 2-fold benefit over time, consistent with defect-mediated electrolyte ingress. SEM morphologies further corroborate these trends, confirming the superior structural stability of Gr_Ni under extended alkaline immersion.

## 1. Introduction

Highly caustic environments are experienced in several chemical engineering processes, such as chlor-alkali production, alumina processing, pulp and paper digestion, alkaline cleaning circuits, and chemical synthesis, where NaOH is either produced or consumed at large scales. Nickel, due to its specific characteristic of rapidly forming a highly passivating corrosion film, is commonly employed in the construction of components exposed to highly caustic environments [1,2]. Material selection is dictated by corrosion risk and uptime requirements. The hydrated Ni(OH)_2_/NiOOH film that develops under such conditions considerably ameliorates but does not arrest the charge transfer [3], thereby allowing the corrosion product film to grow, though slowly, which eventually becomes a concern over long service life [4]. Therefore, a surface modification that can considerably slow down or stop the development and growth of the corrosion product film on the Ni surface has significant commercial value.

Layer(s) of quality graphene on metals can provide a remarkable and durable barrier against corrosion [5]. The reasons are: graphene with minimal defects is effectively impermeable even to small atomic entities (e.g., He), as well as it possesses robust chemical inertness and toughness. The unique combination of these attributes qualifies graphene to be an ideal coating material for corrosion resistance of metallic substrates [6,7,8,9]. To elaborate, graphene’s hexagonal lattice of sp^2^-bonded carbon presents a geometric pore size of ≈0.064 nm [10], which is too narrow to allow ingress of common corrosives (e.g., chloride, oxygen, and hydroxide) that are too large in size. In practice, however, graphene grown on metals invariably contains structural imperfections, most notably interdomain (grain) boundaries/discontinuities, that are pathways for electrolyte permeation [11], thereby critically undermining graphene’s ability as an effective barrier. The deleterious effect of discontinuities in the graphene layer was demonstrated by Prasai et al. [12], who compared coatings developed by chemical vapor deposition (CVD) and by mechanically transferring multilayers on Ni. CVD graphene yielded a 10-fold improvement in corrosion resistance, whereas the improvement due to 2–4 layers of transferred graphene stacks was, at most, 4-fold (since the latter possessed far greater discontinuities). This divergence reflects fundamental differences in film genesis and interface quality.

Despite these advances, relatively few studies have examined graphene-coated metals under prolonged immersion in strongly alkaline solutions [5]. Most prior investigations were limited to short-term exposure durations or were conducted in neutral and acidic electrolytes, leaving the long-term performance in caustic environments largely unknown. This knowledge gap is particularly relevant for the aforementioned industries, where components must withstand caustic conditions over extended service lifetimes. With the background of the demonstrated ability of multilayer graphene (MLG) to be a more effective corrosion barrier and the significant industrial need to ameliorate caustic corrosion, this study investigates the ability of MLG on Ni to improve the metal’s resistance to caustic corrosion in a highly alkaline environment (0.5 M NaOH) for extended durations (>80 days). To establish the profound role of the robustness of graphene, this study also includes tests using less robust and/or defective graphene, which was intentionally developed by tailoring CVD parameters.

## 2. Materials and Methods

### 2.1. Substrate Preparation

Graphene films were synthesized on coupons (15 × 15 × 1 mm^3^) sectioned from a high-purity nickel foil (procured from Puratronic^®^ 99.9945% metals basis, Alfa Aesar, Ward Hill, MA, USA). The coupons were sequentially ground using silicon carbide abrasive papers of grit sizes P320, P800, P1200, and P2500 for 4 min each; followed by polishing with a 3 µm diamond suspension for 2 min. The samples were then ultrasonically cleaned in ethanol for 10 min, rinsed with deionized water, and dried under a stream of dry compressed air.

### 2.2. Graphene Growth via LPCVD

The cleaned Ni coupons were placed in a low-pressure CVD (LPCVD) system equipped with a horizontal quartz tube furnace with 23 mm inner diameter; (Agilent Technologies, London, UK). To control the interaction with the incoming gas flow, custom-made ceramic holders oriented the substrates at a 33° tilt relative to the inlet direction. The quartz tube was evacuated to ~10 mTorr using a dry scroll pump (nXDS10i, Edwards, München, Germany), after which, a flow of Ar/H_2_ (85/15 vol%) at 250 sccm was introduced to reach a pressure of 1.18 Torr. The substrates were annealed at 1070 °C for 40 min under this Ar/H_2_ atmosphere. Subsequently, n-hexane vapor (1 sccm) was introduced as the carbon precursor, increasing the total pressure to 1.23 Torr (corresponding to an n-hexane partial pressure of ~0.05 Torr). Graphene was grown for 60 minutes under these conditions, after which, the furnace was cooled to below 100 °C under Ar/H_2_ flow to prevent oxidation [13].

### 2.3. Characterization of Graphene

Raman spectroscopy was utilized to confirm graphene formation, assess layer thickness, and quantify defect density. Analyses were performed using a Renishaw inVia Raman Microscope (Renishaw plc, Wotton-under-Edge, UK) equipped with a 514 nm (green) excitation laser (17.8 mW). Spectra were acquired in extended mode with a scan range of 100 to 4000 Raman shift (cm^−1^). The exposure time was 10 s with a single accumulation per spectrum. An Olympus 50X long working distance lens (8 mm focal length) was employed with a spot size of ~3 µm.

Surface morphology and coating uniformity were examined using a Phenom XL benchtop scanning electron microscope (Thermo Fisher Scientific, Waltham, MA, USA) operated at 10 kV in secondary electron mode. SEM imaging was conducted both before and after electrochemical testing to analyze changes in surface features and assess the integrity of the graphene coatings post-corrosion exposure.

### 2.4. Characterization of Corrosion Resistance

Electrochemical measurements were carried out using a Princeton Applied Research potentiostat (PARSTAT 2273, AMETEK, Oak Ridge, TN, USA) in a standard three-electrode configuration to assess the corrosion performance of graphene-coated and uncoated nickel specimens. A saturated calomel electrode (SCE) was employed as the reference electrode, while a platinum mesh served as the counter electrode, and the test sample acted as the working electrode with an exposed surface area of 0.785 cm^2^. All experiments were conducted in a 0.5 M NaOH (pH ≈ 13.7) solution at ambient temperature to simulate a highly aggressive corrosive environment.

Prior to electrochemical measurements, all specimens were stabilized by immersion at open circuit potential (OCP) for 2 h, ensuring that the potential drift was restricted within ±10 mV. Potentiodynamic polarization (PDP) experiments were subsequently carried out starting from −250 mV relative to the stabilized OCP and extending anodically up to +500 mV vs OCP at a scan rate of 0.5 mV·s^−1^. Electrochemical impedance spectroscopy (EIS) measurements were performed over a frequency range of 1 MHz to 10 mHz using a 10 mV AC perturbation applied at OCP. To minimize artifacts, impedance spectra were primarily analyzed between 10 kHz and 10 mHz, thereby avoiding high-frequency inductive contributions and low-frequency signal distortion. Each experiment was repeated at least three times on independently prepared specimens to confirm reproducibility and statistical reliability. In addition, corrosion current (*I_corr_*) and corrosion potential (*E_corr_*) were extracted from the PDP curve using EC-Lab V11.50 software.

## 3. Results and Discussion

### 3.1. Raman Spectroscopic Evaluation of Multilayer Graphene Films

**Figure 1:**
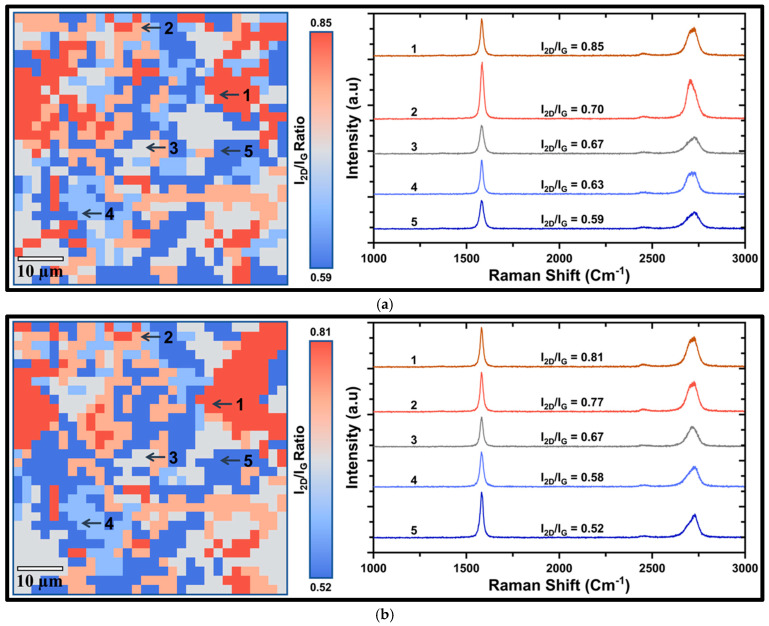
Spatial Raman map (I_2D_/I_G_) showing the distribution of graphene layer number across a 60 × 60 μm^2^ substrate area (left) before immersion in 0.5 M NaOH and the corresponding Raman spectra (right) highlighting the I_2D_ and I_G_ peaks along with their intensity ratios across the substrate for (**a**) Gr_Ni_DF and (**b**) Gr_Ni.

Raman mapping (over 60 × 60 µm^2^) in conjunction with the point spectra at presents layer distribution and lateral uniformity of the coatings before exposure to 0.5 M NaOH. For less robust or highly defective graphene coatings, Gr_Ni_DF (Figure 1a), the map shows pronounced contrast and a broader distribution of I_2D_/I_G_ ratio (0.59–0.85), inferring substantial lateral variability in the number of layers and coating thickness. The negligible D peak intensity across all spectra suggests a relatively low basal-plane defect density. The domains spanning from a few layers (I_2D_/I_G_: 0.85) to thicker multilayer (I_2D_/I_G_: 0.59) suggest that the graphene coating on Gr_Ni_DF is less robust. In contrast, Gr_Ni (Figure 1b) possesses a markedly consistent spectra, with I_2D_/I_G_ ratios ranging between 0.52 and 0.81, suggesting uniform and robust coating across the surface.

#### 3.1.1. Graphene Coating on Ni for Corrosion Protection in Alkaline Media

**Figure 2:**
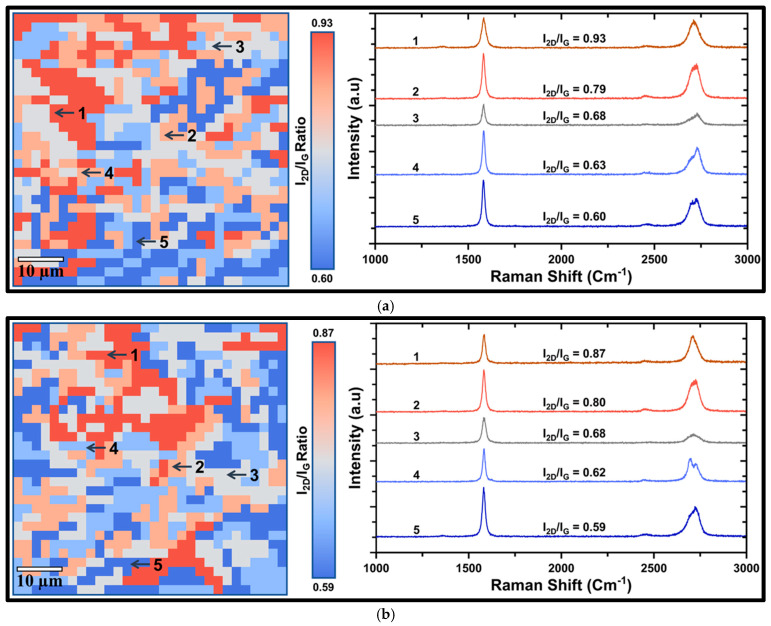
Spatial Raman map (I_2D_/I_G_) showing the distribution of graphene layer number across a 60 × 60 μm^2^ substrate area (left) after a 4-day immersion in 0.5 M NaOH and the corresponding Raman spectra (right) highlighting the I_2D_ and I_G_ peaks along with their intensity ratios across the substrate for (**a**) Gr_Ni_DF (3–4 layers) and (**b**) Gr_Ni (5–7 layers).

Raman mapping and spectra after 4 days of exposure to 0.5 M NaOH for Ni coated with relatively defective graphene (Gr_Ni_DF) suggest considerable deterioration in the number of layers of graphene, as evident from an increase in I_2D_/I_G_ ratio range to 0.60–0.93 (Figure 2a) from 0.59–0.85 prior to exposure (Figure 1a). The number of layers decreased to ≈3 layers (as suggested by the I_2D_/I_G_ ratio, 0.93) at the locations of less robust graphene (as indicated in Figure 2a) that suffered enhanced alkaline wetting and deterioration [8], whereas areas of robust graphene (5–7 layers) resisted such deterioration. Such robust graphene (5–7 layers) occupied most of the surface of Gr_Ni (as indicated in Figure 1b), and hence, the surface of Gr_Ni suffered considerably less deterioration after 4 days of exposure to 0.5 M NaOH, as suggested in Raman mapping and spectra (Figure 2b).

#### 3.1.2. Corrosion Resistance Under Imposed Aggressive Electrochemical Conditions

**Figure 3:**
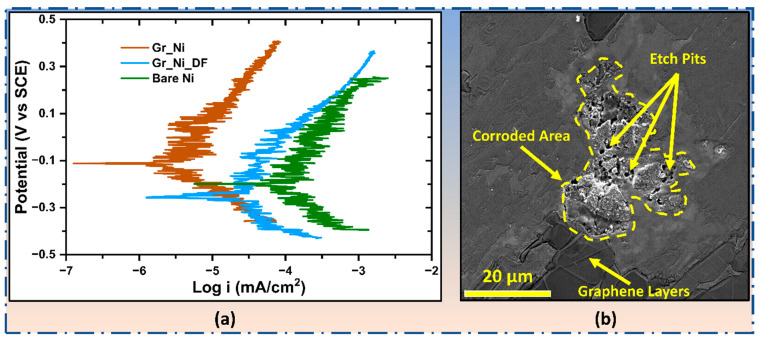
(**a**) PDP curves for Gr_Ni, Gr_Ni_DF, and bare Ni in 0.5 M NaOH (after 2 h immersion) and (**b**) SEM image of isolated localized corrosion sites observed on the Gr_Ni surface after PDP testing, while the surrounding areas largely retain an intact graphene coating.

Potentiodynamic polarization (PDP) was carried out on Gr_Ni, along with Gr_Ni_DF and uncoated Ni, to examine the extent of enhanced corrosion resistance conferred by graphene coating. It is worthwhile to note that PDP assesses corrosion resistance under electrochemically aggressive conditions. During PDP, a working electrode (graphene-coated sample) is also subjected to overpotentials in anodic regime, which constitutes evaluation of the test material’s, including coating’s, ability/integrity to withstand forced/imposed electrochemical conditions. PDP curves for Gr_Ni, Gr_Ni_DF, and uncoated Ni (Figure 3a) determined electrochemical susceptibility to corrosion (*E_corr_*, electrochemical potential, and OCP) and the rate of corrosion (*I_corr_*, corrosion current density) in 0.5 M NaOH after 2 h of immersion in the same solution. Ni with a robust coating, Gr_Ni (as established in Figure 1b and Figure 2b), showed nearly two orders of magnitude superior resistance than bare Ni (Figure 3a), as evidenced by the values of *I_corr_*, i.e., 4.0 × 10^−3^ μA for Gr_Ni (*cf.*, 1.66 × 10^−1^ μA for bare Ni). The less robust coating, Gr_Ni_DF, improved the resistance only by an order of magnitude (*I_corr_*, 3.2 × 10^−2^ μA). Notably, the PDP curves in Figure 3a are appreciably more serrated than those in the reported studies on graphene-coated metals in acidic [14,15] or neutral salt [16,17] electrolytes. The fundamental reason is that the current densities in this study were in a more sensitive regime (as low as 10^−3^ μA·cm^−2^), where even a minor fluctuation is picked up. However, such fluctuations may also be attributed to (i) rapid formation and phase transitions (Ni(OH)_2_/NiOOH) during the potential sweep (which impose stepwise changes in interfacial capacitance and charge-transfer kinetics and thus drive current fluctuations [18]) and (ii) the onset of oxygen evolution together with intermittent hydroxide precipitation/rupture at defects, causing bubble adhesion/detachment and local current/resistance variations that periodically occlude active area [19]. Localized corrosion of the Gr_Ni surface following the destructive PDP testing is evidenced by the formation of isolated etch pits and disrupted film morphology, as shown in the representative SEM image in Figure 3b.

#### 3.1.3. Corrosion Resistance Under Unimposed Electrochemical Condition

**Figure 4:**
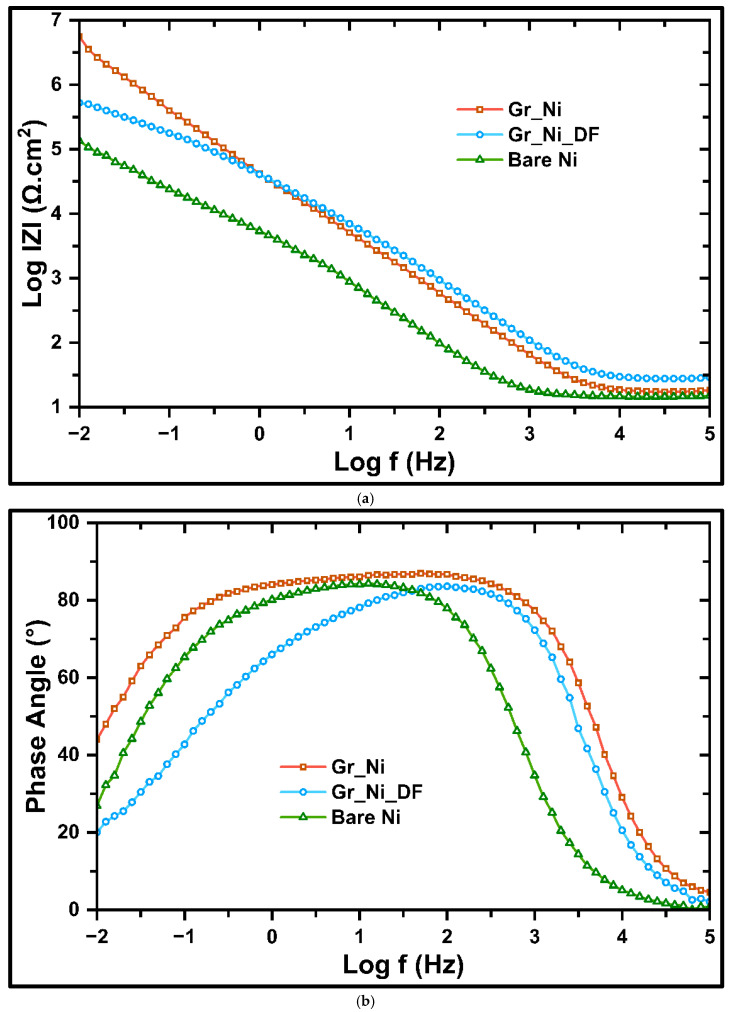
EIS of Gr_Ni, Gr_Ni_DF and bare Ni in 0.5 M NaOH (after 2 h immersion): (**a**) Bode impedance plots and (**b**) Bode phase plots.

Unlike PDP, electrochemical impedance spectroscopy (EIS) is a non-destructive technique since it perturbs the system with a very small AC voltage (typically 5–10 mV) rather than driving it to corrosion or passivation. The EIS results, presented in Figure 4a and Figure 4b, compare mechanistic insights into the corrosion resistance of Gr_Ni, Gr_Ni_DF, and bare Ni in 0.5 M NaOH (after 2 h of immersion in the same solution). The impedance (|Z|) at the lowest frequency, which is a broad measure of corrosion resistance, is nearly two orders of magnitude greater for Gr_Ni than for bare Ni (Figure 4a), whereas that for Gr_Ni_DF is less than an order of magnitude greater. At high frequencies (10^3^–10^5^ Hz), all samples converge toward the solution-controlled resistance, but subtle differences emerge in the interfacial capacitance response: Gr_Ni_DF shows slightly higher |Z|, suggesting a lower effective coating capacitance, while Gr_Ni demonstrates a uniform capacitive barrier. The mid-frequency regime (10^0^–10^3^ Hz) presents a long, near-linear decline in |Z| for Gr_Ni and Gr_Ni_DF, indicative of near-ideal capacitive behavior at the coating/electrolyte interface. In contrast, bare Ni deviates sharply due to its porous Ni(OH)_2_/NiOOH passive film and higher charge-transfer leakage over the entire surface. The most significant difference arises in the low frequency regime (10^−2^–10^0^ Hz), which is a broad measure of corrosion resistance, suggesting that Gr_Ni shows >1.8 orders of magnitude higher corrosion resistance than bare Ni. The less robust Gr_Ni_DF shows only 0.5 orders of magnitude higher resistance. It is worthwhile to note that the trend of corrosion resistance determined by EIS is consistent with the trend in *I_corr_* for these samples in PDP results (Figure 3a).

The Bode phase spectra (Figure 3b), while substantiating the trend in the preceding discussion, also provide further insights into the dielectric and interfacial characteristics of the graphene-coated Ni compared to bare Ni. As seen in the spectrum, the phase angle of Ni with robust graphene coating (Gr_Ni) rapidly increases with decreasing frequency, attaining a broad and well-defined plateau in the range of ~88°, which extends across the low/mid-to-high frequency regime (10^−1^–10^3^). The close-to 90° peak phase angle and the wide plateau are the characteristics of a nearly ideal capacitive response, where both the coating/electrolyte and metal/electrolyte interfaces act in unison to suppress charge transfer, demonstrating the presence of a continuous and highly resistive barrier. On the other hand, the less robust graphene (Gr_Ni_DF) attains a similar but lower peak phase angle (~80°) and a narrower plateau (ranges: 10^2^–10^3^ frequency), rolling off earlier at both high frequencies and mid-frequencies. These highlight the detrimental impact of non-uniform coverage and interconnected defect pathways, which compromise barrier efficiency over extended immersion. Despite the absence of a barrier coating, the bare Ni exhibits a reasonably high phase angle (~82°), which may be attributed to the highly passivating characteristic of Ni. However, the considerably less wide plateau (limited to the mid-frequencies, 10^0^–10^2^) is attributed to the formation of a native Ni(OH)_2_/NiOOH passive layer that provides only partial charge-transfer resistance. The comparative findings from the Bode impedance (Figure 4a) and phase (Figure 4b) spectra infer that, while the native hydroxide film and less robust graphene provided some protection, the robust, uniform, multilayer graphene coating (Gr_Ni) ensures an extended capacitive regime, conferring the ability to effectively retard hydroxide ingress and electrochemical integrity in an aggressive NaOH medium.

### 3.2. Evaluation of Durability of Corrosion Resistance Due to Graphene Coating

#### 3.2.1. Electrochemical Evaluation During Long-Term Exposure to NaOH

**Figure 5:**
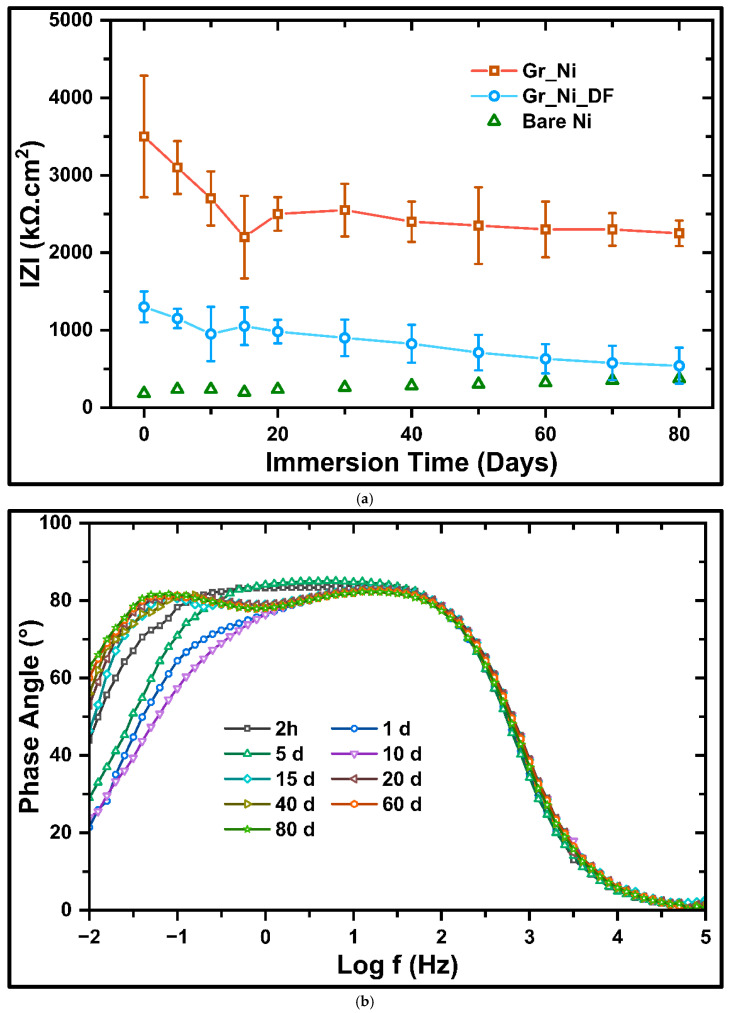

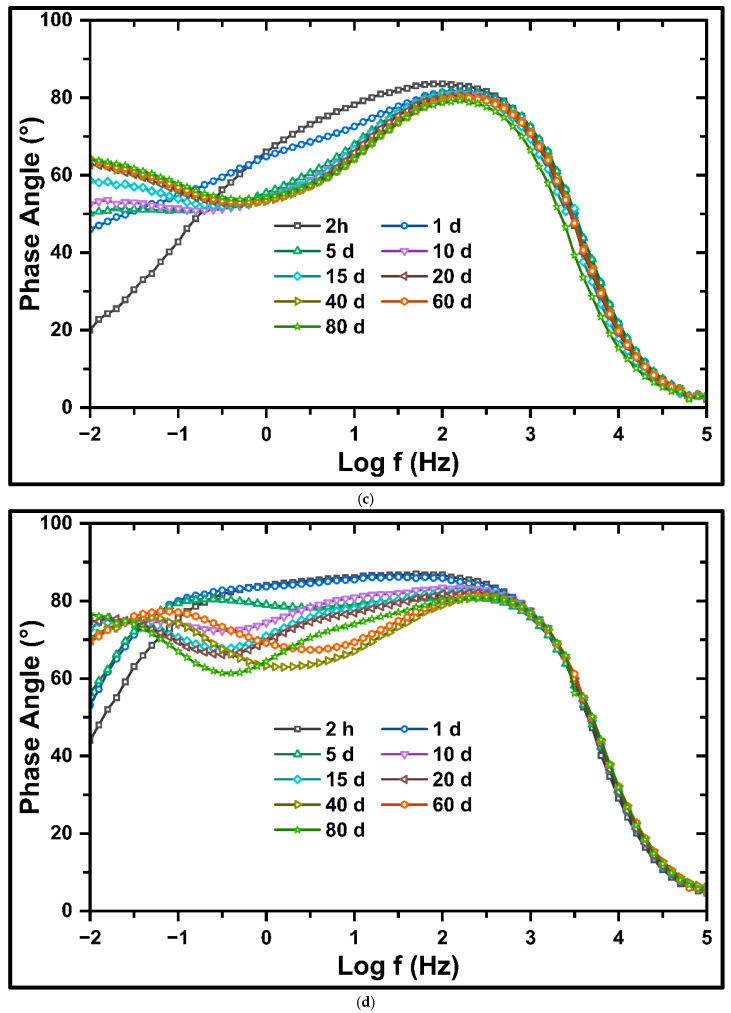
(**a**) Impedance spectra of bare Ni, Gr_Ni_DF, and Gr_Ni after immersion in 0.5 M NaOH for up to 80 days. Corresponding Bode phase angle plots after equivalent immersion durations for (**b**) bare Ni, (**c**) Gr_Ni_DF, and (**d**) Gr_Ni.

Long-term durability of the corrosion resistance of Ni conferred due to a robust graphene coating (Gr_Ni) in 0.5 M NaOH was quantified by monitoring the area-normalized low-frequency impedance modulus |Z| at OCP for durations up to 80 days, as depicted in Figure 5a; error bars further denote statistical robustness of the data. Gr_Ni shows a remarkably greater |Z|, ~3500 kΩ·cm^2^ (*cf.*, Gr_Ni_DF and bare Ni), at the beginning of the exposure. The impressively higher |Z|, which is attributed to the compact multilayer film imposing large pore resistance and a near ideal capacitive behavior (as discussed earlier), is maintained throughout the long exposure for 80 days. However, the transient drop in |Z| during the first 10–15 days is attributed to some electrolyte ingress through occasional locations of defects that are eventually plugged by reaction products, as well as, it can be attributed to relaxation of entrained species and redistribution of ions within the coating microstructure. After this period, a broad quasi-steady plateau is observed, signifying long-time sealing against OH^−^ transport. On the other hand, Gr_Ni_DF starts at substantially lower |Z| (1400 kΩ·cm^2^), a behavior characteristic of percolation through non-uniform regions, i.e., pinholes, grain boundaries, and micro-cracks, which progressively lower pore resistance and increase faradaic leakage at low frequency; |Z| decays monotonically to ~600 kΩ·cm^2^ by ~80 days. Bare Ni maintains the smallest |Z| throughout the immersion time, with only a gradual upward drift attributed to thickening and reorganization of the hydroxide layer (*cf*., Figure 4), whose mixed conduction offers limited blocking in strong alkali.

Figure 5b–d present the temporal evolution of the Bode phase angle for bare Ni, Gr_Ni_DF, and Gr_Ni, respectively, during immersion in 0.5 M NaOH for durations up to 80 days. For bare Ni, the response is dominated by a single, relatively narrow plateau at low-to-mid frequency (10^0^–10^2^) that corresponds to the charge transfer phenomena at the metal/alkaline electrolyte interface. The rapid decay in the early stages (~10 days) at the low frequencies reveals early activation of ohmic and faradaic pathways. As the immersion time exceeds 10 days, the peak broadens and shifts subtly toward lower frequencies, while the low-frequency phase rises modestly, consistent with growth of a porous hydroxide layer, introducing an additional but sluggish capacitive contribution without conferring ideal passivity.

For Gr_Ni_DF, the phase spectra during early stages (2 h to 1 day) exhibit overlapping high- and mid-frequency features from the coating/electrolyte and metal/electrolyte interfaces. As immersion proceeds (5–20 days), the merged feature begins to separate into distinct responses; the limb in the low frequency regime progressively becomes less capacitive, indicating the increasing contribution of defects. After 20 days, the phase rises at the low frequency due to occlusion by corrosion products inside pores, which adds a slow capacitive element but does not broaden the mid-band plateau. The progressive narrowing and eventual degradation of the low-frequency phase plateau directly correlate with the monotonic decrease in |Z| (Figure 5a), signifying increasing electrolyte percolation through interconnected defects rather than isolated pitting or delamination.

Gr_Ni maintains the broadest and highest plateau (~85–90°) from low to mid frequency (10^−1^–10^3^) over the entire 80 days of exposure, with only minor early shifts attributable to wetting/relaxation within near-surface microdefects. The absence or considerably less prominence of a mid-band depression and sustained broad plateau signifies a near-ideal capacitive coating response, while low-frequency decay remains delayed, indicating limited activation of Faradaic pathways. In this context, the persistence of a high phase angle in a low-frequency regime and stable plateau is indicative of effective long-term barrier integrity, consistent with prior studies on passive film evolution in strongly alkaline environments, where sustained capacitive plateaus have been associated with compact, stable interfacial films and enhanced corrosion resistance [20].

Nyquist plots for the bare and graphine-coated Ni samples immersed in 0.5 M NaOH for 2 h, 20 d, 40 d and 80 d (Figure S1) corroborate trends of durability as assessed from the Bode plots (Figure 5b–d). Bare Ni exhibits a depressed capacitive semicircle with comparatively smaller diameter (Figure S1a), which is characteristic of charge-transfer-controlled corrosion at the metal/environment interface. The modest rightward shift of the intercept with increasing exposure time (20 d–80 d) indicates gradual growth of surface hydroxide species (Ni(OH)_2_/NiOOH), which causes a light increase in polarization resistance but without any robust barrier stabilization. Gr_Ni_DF displays intermediate arc diameters and stronger semicircle depression, reflecting surface heterogeneity and distributed time constants associated with electrolyte penetration through defective graphene regions (Figure S1b). In contrast, Gr_Ni (Figure S1c) consistently displays the largest capacitive arcs (*cf.*, bare Ni and Gr_Ni_DF) across all immersion durations, demonstrating considerably enhanced polarization resistance and suppressed charge-transfer kinetics. The persistence of high impedance (large arc diameter) for Gr_Ni is consistent with the great low-frequency |Z| and broad near-capacitive phase plateau, as demonstrated in Figure 5, confirming long-term resistance to OH^−^ transport.

**Figure 6:**
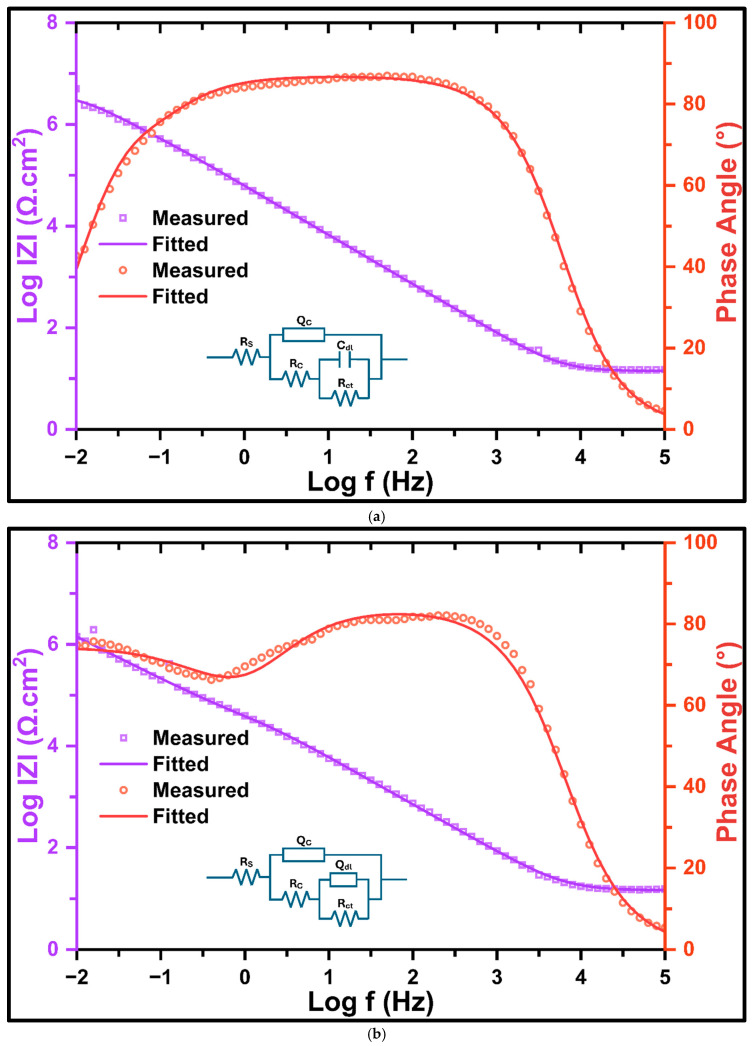

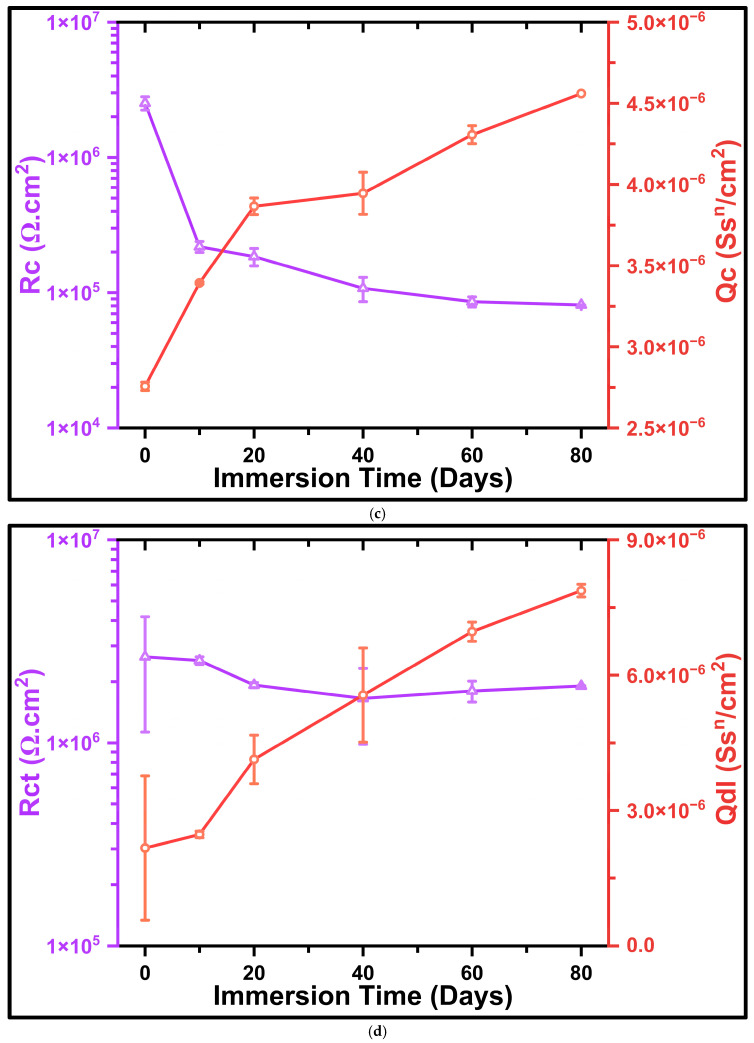
Typical Bode impedance magnitude and phase-angle plots showing experimental EIS data and corresponding EEC-fitted spectra for graphene-coated Ni (Gr_Ni) in 0.5 M NaOH after (**a**) 2 h and (**b**) 20 days of immersion. Evolution of EEC-fitted parameters for graphene-coated Ni in 0.5 M NaOH up to 80 days: (**c**) coating resistance (Rc) + coating capacitance (Qc) and (**d**) charge-transfer resistance (Rct) + double layer capacitance (Qdl), based on the proposed EEC, for up to 80 days of immersion in 0.5 M NaOH.

The experimental EIS spectra of graphene-coated nickel (Gr_Ni) immersed in 0.5 M NaOH were quantitatively fitted using the equivalent electrical circuits schematically embedded in the corresponding Bode plots (Figure 6a,b). For short exposure (2 h, Figure 6a), the impedance response is described by the solution resistance (Rs) in series with a coating branch consisting of the coating resistance (Rc) in parallel with the coating constant phase element (Qc), followed by an interfacial branch comprising the charge-transfer resistance (Rct) in parallel with the double-layer capacitance (Cdl). This configuration indicates an intact coating barrier and near-ideal capacitive behavior of the buried interface at the early stage of alkaline exposure. After prolonged immersion (20 days, Figure 6b), Cdl is replaced by a constant phase element (Qdl), reflecting non-ideal capacitive behavior associated with increasing interfacial heterogeneity and distributed electrochemical activity beneath the graphene coating. In both cases, the fitted spectra exhibit excellent agreement with the experimental impedance magnitude and phase-angle data over the full frequency range, confirming the suitability of the selected EEC models for describing the time-dependent electrochemical response of the Gr_Ni system in alkaline media.

Figure 6c,d summarize the time-dependent evolution of the EEC-derived coating and interfacial parameters for Gr_Ni during immersion in 0.5 M NaOH. As shown in Figure 6c, the coating resistance (Rc) decreases sharply from 2 h to 10 days (approximately by an order of magnitude), consistent with rapid electrolyte uptake and activation of ionic transport pathways through coating defects. Thereafter, Rc declines more gradually from 10 to 40 days and approaches a near-steady trend up to 80 days, suggesting that further electrolyte penetration becomes progressively limited, plausibly due to the development of tortuous transport pathways and partial blockage by corrosion products within the accessible defects. In parallel, the coating CPE magnitude (Qc) increases monotonically with immersion time, indicating a progressive increase in the effective coating capacitance associated with electrolyte uptake and an enhanced dielectric response. The interfacial parameters (Figure 6d) show a modest reduction in charge-transfer resistance (Rct) during the early-intermediate stages, followed by stabilization, whereas the double-layer CPE (Qdl) increases continuously with time. The concurrent increase in Qdl and stabilization of Rct is indicative of an increasingly heterogeneous buried interface beneath the graphene coating, with distributed electrochemical activity developing during long-term alkaline exposure.

#### 3.2.2. Morphological Evaluation During Extended Exposure to NaOH

**Figure 7:**
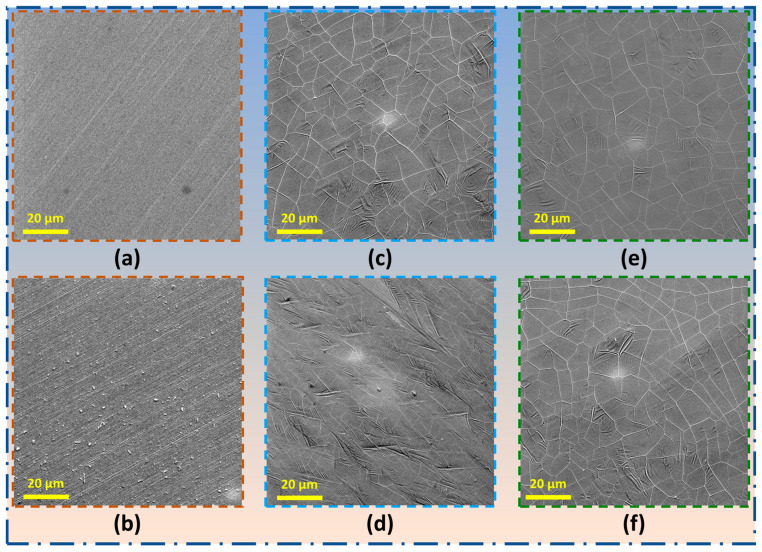
SEM images of (**a**,**c**,**e**) bare Ni, Gr_Ni_DF, and Gr_Ni, respectively, before immersion and (**b**,**d**,**f**) bare Ni, Gr_Ni_DF, and Gr_Ni, respectively, after immersion in 0.5 M NaOH for 4 days.

SEM topographs in Figure 7 compare morphologies of bare Ni, Gr_Ni_DF, and Gr_Ni before immersion (Figure 7a, Figure 7c and Figure 7e repectively) and after 4 days of exposure to 0.5 M NaOH (Figure 7b, Figure 7d and Figure 7f respectively), highlighting the distinct influence of graphene quality on surface stability. Prior to immersion, the bare Ni surface (Figure 7a) presents a relatively smooth, mechanically polished topography with faint grinding traces. Alkaline exposure markedly roughens the surface (Figure 7b) as it develops heterogeneous etch pits, pronounced grain-boundary grooving, and dispersed bright particulates, presumably of Ni(OH)_2_/NiOOH precipitates, evidencing non-uniform dissolution/passivation. The less robust graphene coating, Gr_Ni_DF (Figure 7c), initially shows a typical multilayer topography of intersecting wrinkles, domain boundaries, and occasional micro-discontinuities from CVD growth/strain relaxation. Upon immersion, these features intensify into a ridge–valley texture with localized tearing and under film nodule formation; bright deposits cluster along wrinkles and seam intersections, consistent with preferential OH^−^ transport and corrosion-product occlusion at percolating pathways, while adjacent terraces exhibit ripple-like micro-delamination (Figure 7d). In contrast, the uniform multilayer film Gr_Ni (Figure 7e) presents a conformal surface with relatively fewer wrinkles and no obvious features of disruptions. After 4 days of immersion (Figure 7f), the film largely retains its continuity, and the surface does not possess any obvious disruptions/pits.

**Figure 8:**
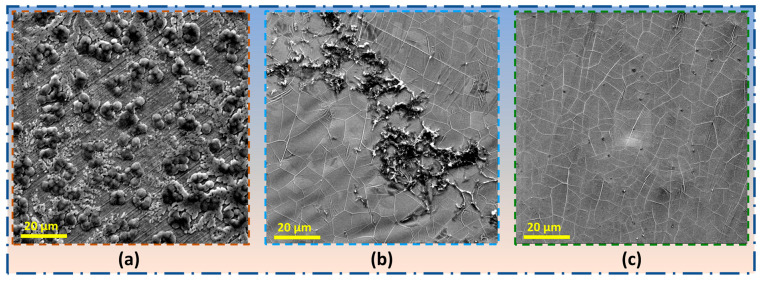
SEM images after 80 days of immersion in 0.5 M NaOH: (**a**) bare Ni, (**b**) Gr_Ni_DF, and (**c**) Gr_Ni.

Figure 8a–c shows the corresponding SEM topographs of bare Ni, Gr_Ni_DF, and Gr_Ni, respectively, after 80 days of immersion in 0.5 M NaOH. Corrosion products on the bare Ni surface (Figure 8a) have transformed into a porous, sponge-like structure with coalesced etch pits and hydroxide deposits because of sustained dissolution in the absence of a barrier. In contrast, upon the 80-day exposure, Ni with less robust graphene coating, i.e., Gr_Ni_DF (Figure 8b), retained graphene on a considerable fraction of the surface, but the surface also has locations of severe corrosion attack (Figure 8b), presumably due to accentuation of the localized corrosion attack in the earlier stages (e.g., Figure 7d) at the disruptions in the coating or at the locations of less robust graphene. In contrast, Ni with uniformly robust graphene (Gr_Ni) possesses no obvious disruptions or corrosion attack, even after 80 days of exposure (Figure 8c).

## 4. Conclusions

This study establishes the long-term corrosion protection capability of CVD-grown multilayer graphene on Ni in strong alkaline media (0.5 M NaOH) for >80 days of immersion. The important findings of this work are as follows.

Robust graphene over Ni (Gr_Ni) exhibits tightly distributed I_2D_/I_G_ ratios before and after immersion, confirming a robust coating with 5–7 layers, whereas the less robust coating (Gr_Ni_DF) shows broader Raman distributions, revealing a coating with inferior uniformity and defects.

Gr_Ni delivers the lowest *I_corr_*, ≈1.8 orders of magnitude lower than bare Ni and maintains superior durability, with impedance values 1.3–1.5 orders higher after 80 days, whereas Gr_Ni_DF shows only a modest reduction in *I_corr_* and a progressive decay in impedance due to defect-mediated transport.

SEM reveals Gr_Ni maintains film continuity with only sparse spots of localized attack after prolonged immersion, while Gr_Ni_DF undergoes pronounced attack, and bare Ni develops a considerably porous corrosion layer and pits.

## Data Availability

Data is contained within the article.

## Supplementary Materials

The following supporting information can be downloaded at: https://www.mdpi.com/article/10.3390/nano16040265/s1, Figure S1: Nyquist plots after immersion in 0.5 M NaOH for up to 80 days for (a) Bare Ni, (b) Gr_Ni_DF, and (c) Gr_Ni
