# Supplementary material for: Graphene Coatings for Durable and Robust Resistance to Caustic Corrosion of Nickel
Source: Nanomaterials (Basel). 2026 Feb 18;16(4):265. doi: 10.3390/nano16040265 (PMC12943579; doi:10.3390/nano16040265)
Supplement: Supplementary file 1 [file nanomaterials-16-00265-s001.zip › nanomaterials-4089643-supplementary.pdf]

# Graphene Coatings for Durable and Robust Resistance to Caustic Corrosion of Nickel

Tanuj Joshi[1], R. K. Singh Raman [1,*], Yiannis Ventikos[1], Saad Al-Saadi[2], and Anthoney De Girolamo[2]

[1]Department of Mechanical and Aerospace Engineering, Monash University, Clayton 3800, Australia

[2]Department of Chemical and Biological Engineering, Monash University, Clayton 3800, Australia

*Corresponding Author: Raman.Singh@monash.edu

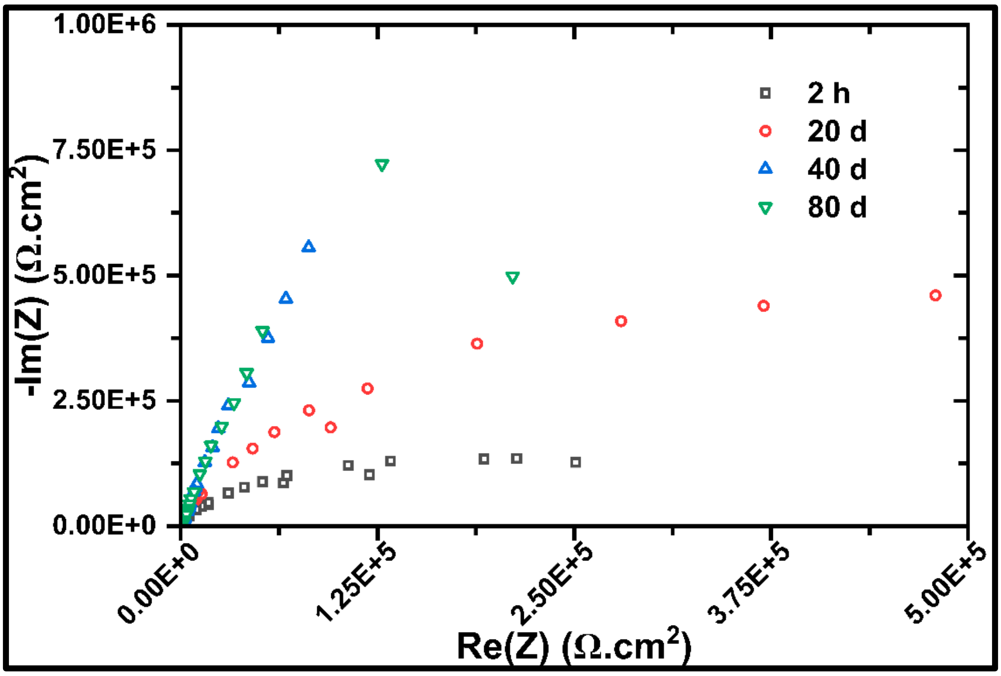


**(a)**

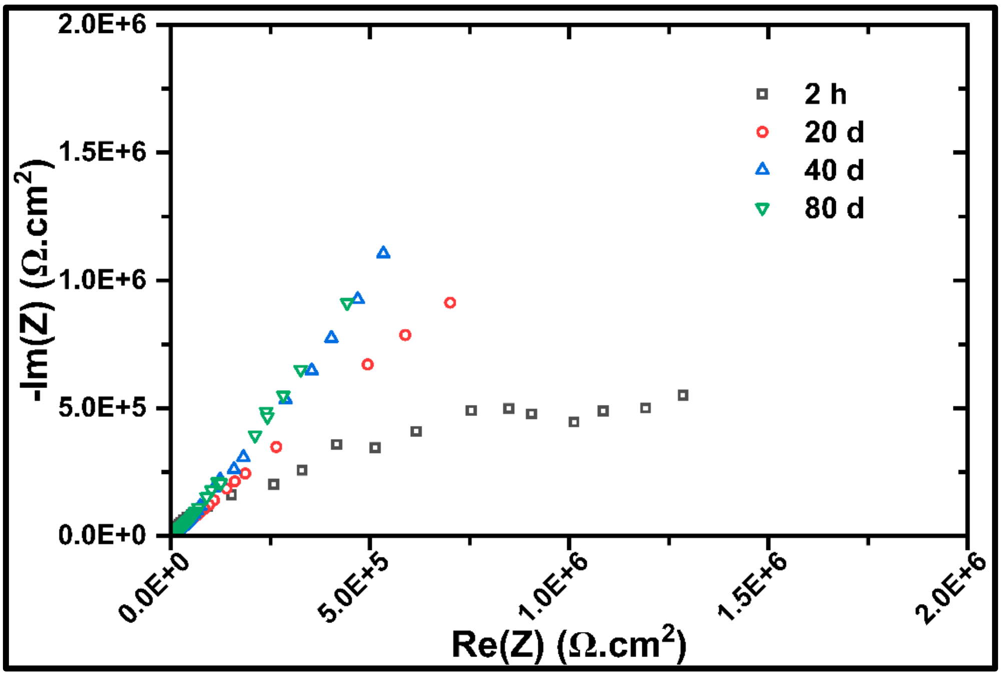


(b)

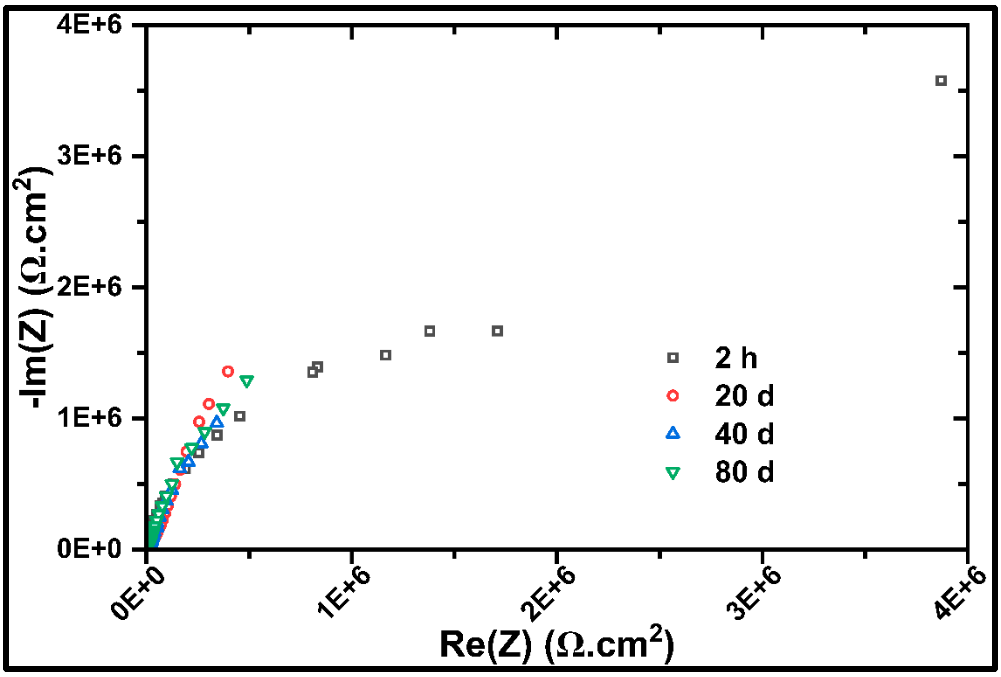


(c)

**Figure S1.** Nyquist plots after immersion in 0.5 M NaOH for up to 80 days for: **(a)** Bare Ni, **(b)** Gr_Ni_DF, and **(c)** Gr_Ni.
